# 35 Burn Prevention in Spanish: Assessment of Content Accuracy, Website Quality, and Readability of Online Sources

**DOI:** 10.1093/jbcr/irad045.009

**Published:** 2023-05-15

**Authors:** Miguel Gonzalez, Pilar Ortega, Justin Gillenwater, Sebastian Vrouwe

**Affiliations:** University of Chicago Pritzker School of Medicine, Chicago, Illinois; University of Illinois College of Medicine; Medical Organization for Latino Advancement, Chicago, Illinois; Keck Medicine of USC, Los Angeles, California; University of Chicago, Chicago, Illinois; University of Chicago Pritzker School of Medicine, Chicago, Illinois; University of Illinois College of Medicine; Medical Organization for Latino Advancement, Chicago, Illinois; Keck Medicine of USC, Los Angeles, California; University of Chicago, Chicago, Illinois; University of Chicago Pritzker School of Medicine, Chicago, Illinois; University of Illinois College of Medicine; Medical Organization for Latino Advancement, Chicago, Illinois; Keck Medicine of USC, Los Angeles, California; University of Chicago, Chicago, Illinois; University of Chicago Pritzker School of Medicine, Chicago, Illinois; University of Illinois College of Medicine; Medical Organization for Latino Advancement, Chicago, Illinois; Keck Medicine of USC, Los Angeles, California; University of Chicago, Chicago, Illinois

## Abstract

**Introduction:**

Many burn injuries are preventable, yet burn prevention information may be inadequate or inaccessible to communities with non-English language preference. Out of the estimated 480,000 people in the US that are treated annually for burns, 14% identify as Latinx. Among the 60 million Latinx individuals living in the US, 33% report limited English proficiency. As a result, many Latinx persons rely on health information in Spanish. The availability and quality of Spanish language information regarding burn prevention has not previously been studied but presents an important opportunity to reduce burn-related injuries for the US Latinx population. Our objective was to systematically analyze the content accuracy, website quality, and readability of Spanish language online information for burn prevention in the home.

**Methods:**

We conducted a search on Google, Bing, and Yahoo using the following key burn prevention terms in Spanish: “prevención de quemaduras,” “prevenir quemaduras,” “evitar quemaduras,” and “impedir quemaduras”. The top 10 results from each search engine and key term were recorded. Using recommendations from national organizations and a burn care expert team, two reviewers independently evaluated the content accuracy of each website and met to reach consensus. We assessed website quality by three major categories based on the Health on the Net Code of Conduct (HONcode): accessibility/usability, credibility, and currency. We scored readability of each website by utilizing five validated readability tests for the Spanish language and generating an average grade reading level needed to understand the article.

**Results:**

After removing duplicates and irrelevant search results, 23 websites in Spanish met inclusion criteria. Across all websites, the content accuracy of information broken down by type of burn was as follows: scald 35%, electrical 28%, contact 20%, and flame 17%. Out of 21 possible points, the top website scored 14 (66.7%) and the average score was 6.6 (31.4%). Using the intraclass correlation coefficients, the interrater reliability was 0.935. For website quality, the average score was 50.2%. The average grade level needed to read the websites was 8.6.

**Conclusions:**

Spanish language online information on burn prevention in the home is often inaccurate and incomplete. Furthermore, overall website quality was lower than 50% according to the HONcode, and the average grade level needed to read the articles is higher than the sixth-grade recommendation from the American Medical Association.

**Applicability of Research to Practice:**

This research highlights the lack of reliable and accessible information in Spanish on home burn prevention. Not only does this research suggest that Spanish-speaking patients should be provided education on burn prevention by their healthcare providers, but more importantly, we propose a call to action to increase the quality of online Spanish materials for burn prevention for the US Latinx community.

